# Development of a nomogram for predicting cancer-specific survival in patients with renal pelvic cancer following surgery

**DOI:** 10.1515/med-2025-1277

**Published:** 2025-09-12

**Authors:** Chung-Cheng Lin, Chao-Yu Hsu

**Affiliations:** Department of Medical Education, Ditmanson Medical Foundation, Chia-Yi Christian Hospital, Chia-Yi, Taiwan; Department of Urology, Puli Christian Hospital, Puli, Taiwan; Department of Artificial Intelligence and Healthcare Management, Central Taiwan University of Science and Technology, Taichung, Taiwan; Department of General Education, National Chin-Yi University of Technology, Taichung, Taiwan

**Keywords:** nomogram, renal pelvic, transitional cell carcinoma

## Abstract

**Objective:**

The aim of this study is to construct a post-operative nomogram for renal pelvic cancer, thereby addressing a gap in the current academic literature and offering a valuable tool for predicting patient outcomes following surgical intervention.

**Methods:**

This study utilized data from the Surveillance, Epidemiology, and End Results program (2004–2017) to analyze patients diagnosed with renal pelvic cancer who underwent surgery. Variables analyzed included demographics, histology, grade, stage, and treatment modalities. Statistical analysis involved Kaplan-Meier and Cox models, developing a nomogram to predict 1-, 3-, and 5-year cancer-specific survival (CSS), validated through receiver operating characteristic (ROC) curves, calibration, and decision curve analysis (DCA) to assess clinical utility.

**Results:**

The training cohort consisted of 1,486 patients, and the validation cohort comprised 637 patients. Factors affecting CSS, analyzed through univariate and multivariate models, included age, histology, cancer grade, stage, and treatment modalities. The developed nomogram, tested via ROC curves and calibration plots, showed robust predictive accuracy for CSS across both cohorts, with its clinical utility demonstrated through DCA.

**Conclusion:**

Age, histology, grade, and stage were significant risk factors for CSS in renal pelvic urothelial carcinoma post-surgery. A nomogram utilizing these factors aids in evidence-based clinical decision-making.

## 1 Introduction

In the earlier study, Yang et al. [1] reported that urothelial carcinoma (UC) of the renal pelvis constitutes approximately 5% of all urothelial tumors and 10% of renal neoplasms. In contrast, tumors located in the ureter are relatively rare, with a ratio to those found in the renal pelvis of approximately 1:3 to 1:4. Subsequently, two studies from Japan published in 1990 discovered that the prevalence of tumors originating from the renal pelvis and the ureter were approximately similar. Tashiro et al. [2] conducted an analysis of 160 patients diagnosed with renal pelvic and ureteral cancer, identifying 71 cases of renal pelvic cancer, 80 cases of ureteral cancer, and 9 cases involving both regions. Consequently, the ratio of renal pelvic cancer to ureteral cancer was determined to be 1:1.3. In another study, Ueda et al. [3] utilized data from the Tokai Urological Cancer Registry, covering the period from 1980 to 1986, to examine the prevalence of renal pelvic and ureteral tumors. In an analysis of 384 cases, 210 patients were diagnosed with renal pelvis cancer and 174 with ureteral cancer, yielding a renal pelvis to ureteral cancer ratio of 1:0.83.

In the study conducted by Van Doeveren et al. [4], an analysis of the period spanning from 1993 to 2017 reveals a significant augmentation in the age-standardized incidence rate of upper urinary tract urothelial carcinoma (UTUC) within the Dutch population, exhibiting a rise exceeding 50%. Specifically, the incidence of UTUC escalated from 2.0 cases per 100,000 person-years in 1993 to 3.2 cases per 100,000 person-years by 2017. Notably, this increment was predominantly observed in instances of UC affecting the ureter, with the estimated annual percentage increase being 2.4% for ureteral carcinoma compared to a 1.5% increase for carcinoma located in the renal pelvis. Yang et al. [1] documented a higher prevalence of UTUC in Taiwan, observed not only within the endemic regions of “blackfoot disease” but also in areas unaffected by this condition. Their research encompassed a comprehensive analysis of 535 consecutive patients diagnosed with pathologically confirmed UC between 1983 and 1998. The findings revealed a distinctive incidence ratio of 1:2.08:6.72 for UC across the renal pelvis, ureter, and urinary bladder, respectively. Contrary to prior studies, the proportion of tumors located in the renal pelvis was identified as the lowest.

Numerous nomograms have been developed to predict outcomes for UTUC, including those for predicting cancer-specific survival (CSS) [5], CSS and overall survival (OS) [6], OS of metastatic UTUC [7], OS following chemotherapy treatment [8], extraurothelial recurrence [9], and to assist in decision-making for endoscopic management [10]. However, there has been a notable scarcity of studies aimed at creating a nomogram specifically for renal pelvis cancer. Although Wang et al. [11] recently published a paper on a prognostic nomogram for CSS, it targeted elderly patients with tumor (T), node (N), and metastasis (M) stage as T1-T3N0M0 cancers and did not focus on predictions after surgery. Therefore, this study endeavors to create a post-operative nomogram for renal pelvic cancer, filling a void in current academic discourse and providing a valuable tool for predicting patient outcomes following surgical intervention.

## 2 Materials and methods

### 2.1 Data source and selection criteria

Data for this study were sourced from the Surveillance, Epidemiology, and End Results (SEER) program, using version 8.4.2. We included records from patients diagnosed with renal pelvic cancer between 2004 and 2017 who underwent surgical treatment. Only patients who received surgical intervention were selected as this provided access to complete pathological reports. Exclusion criteria were applied to enhance the specificity of our study population. We excluded cases with unspecified tumor side, bilateral tumors, unknown grade, and histologies other than transitional cell carcinoma (TCC) or papillary TCC. Additionally, cases with unknown survival status or indeterminate T.N.M. staging were omitted to ensure clarity in staging and outcome measures.

### 2.2 Variables collected

The demographic variables included in our analysis were gender and race (categorized as white and others), along with age groups (<60, 60–69, 70–79, ≥80 years). From a pathological standpoint, we included histology (TCC and papillary TCC), grade (well, moderately, poorly, and undifferentiated), and the American Joint Committee on Cancer stage (7th edition) (based on TNM classification). Treatment modalities such as radiotherapy and chemotherapy were also considered.

### 2.3 Statistical analysis

Statistical analysis was conducted using R software, version 4.3.2. Survival analysis was performed using the Kaplan-Meier method to calculate CSS rates at 1, 3, and 5 years. Differences in survival rates across various factors were assessed. The patient cohort was divided into a training cohort (70%) and a validation cohort (30%) to support the development and validation of prognostic models. In the training cohort, univariate Cox proportional hazards models were used to evaluate the influence of each variable on survival. Variables with a *p*-value less than 0.05 were included in a multivariate Cox regression analysis to identify significant prognostic factors. These factors were used to calculate a concordance index (C-index); a C-index greater than 0.70 was required to utilize these prognostic factors in constructing a nomogram.

### 2.4 Validation of the nomogram

The nomogram was developed to predict 1-, 3-, and 5-year CSS. The reliability of the nomogram was subsequently assessed. Validation involved first applying the model to the training cohort and then to the validation cohort. The validation process included the use of receiver operating characteristic (ROC) curves to evaluate the discrimination ability of the nomogram, calibration to assess the accuracy of the predictions against actual outcomes, and decision curve analysis (DCA) to determine the clinical usefulness of the nomogram by quantifying the net benefits at various threshold probabilities. These methods were chosen to ensure a comprehensive evaluation of the model’s performance and its potential utility in clinical settings.


**Ethics approval:** This study was granted ethical approval by the Ethics Committee of “Ditmanson Medical Foundation Chia-Yi Christian Hospital” (IRB2024043). Given that the patient data utilized was sourced from the anonymized SEER database, the requirement for written informed consent from study participants was waived. All procedures were conducted in strict adherence to pertinent guidelines and regulations. Research involving human participants, human materials, or human data was carried out in compliance with the “Declaration of Helsinki.”

## 3 Results

**Figure 1:**
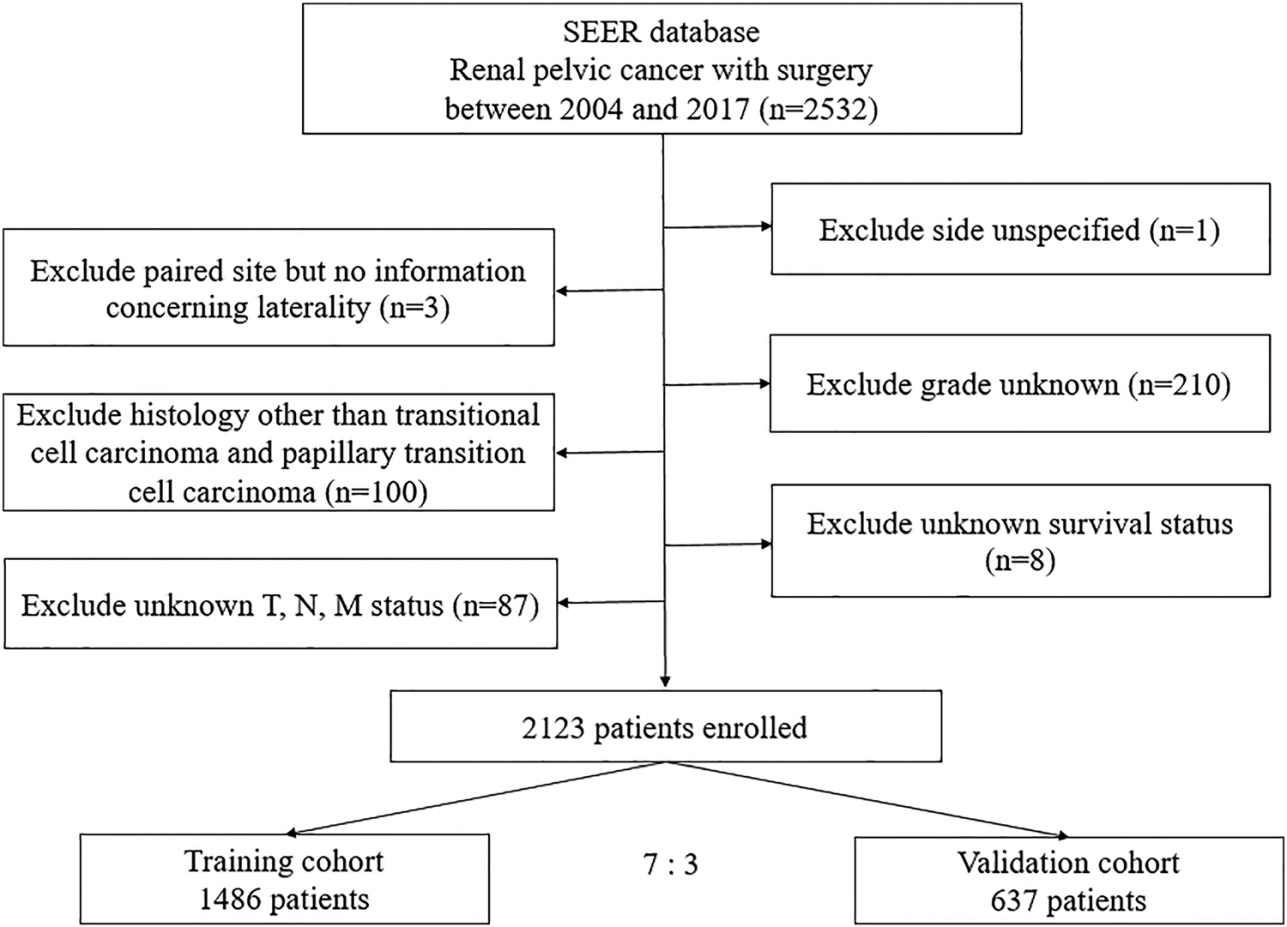
Flowchart of patients’ selection.

The flowchart detailing the selection process of patients is depicted in Figure 1. Table 1 presents the baseline characteristics of patients in both the training and validation cohorts. The training cohort included 1,486 patients, whereas the validation cohort comprised 637 patients. Gender distribution across both cohorts was nearly equivalent, with males representing approximately 58% of each group. The majority of patients in both cohorts were white, accounting for 87.6% in the training cohort and 85.7% in the validation cohort. Age distribution was evenly matched across four age groups: under 60, 60–69, 70–79, and 80 or older, with no significant differences in age distribution between the cohorts. Histologically, patients were diagnosed with either TCC or papillary TCC, with similar distributions in both cohorts. The grading of the tumors ranged from well to undifferentiated, with no significant differences noted. Staging of the cancers also showed consistency across the cohorts, with stages I through IV represented. Treatment modalities indicated that a majority of patients did not receive radiotherapy, while chemotherapy usage was more prevalent in the training cohort compared to the validation cohort, marking the only significant difference between the groups in terms of treatment. Overall, the observed CSS rates at 1-, 3-, and 5-year intervals for all patients were 83.9, 65.9, and 57.4%, respectively.

**Table 1:** Baseline characteristics of the training and validation cohorts

		Training cohort (*n* = 1,486)	Validation cohort (*n* = 637)	*p*
		Number (%)	Number (%)	
Sex	Male	863 (58.1%)	367 (57.6%)	0.8435
	Female	623 (41.9%)	270 (42.4%)	
Race	White	1,302 (87.6%)	546 (85.7%)	0.2313
	Others	184 (12.4%)	91 (14.3%)	
Age	<60	233 (15.7%)	100 (15.7%)	0.9867
	60–69	340 (22.9%)	144 (22.6%)	
	70–79	483 (32.5%)	212 (33.3%)	
	≥80	430 (28.9%)	181 (28.4%)	
Histology	Transitional cell carcinoma	719 (48.4%)	295 (46.3%)	0.3806
	Papillary transitional cell carcinoma	767 (51.6%)	342 (53.7%)	
Grade	Well differentiated	48 (3.2%)	29 (4.6%)	0.3727
	Moderately differentiated	174 (11.7%)	72 (11.3%)	
	Poorly differentiated	343 (23.1%)	157 (24.6%)	
	Undifferentiated	921 (62.0%)	379 (59.5%)	
Stage	I	433 (29.1%)	192 (30.1%)	0.0770
	II	154 (10.4%)	89 (14.0%)	
	III	562 (37.8%)	224 (35.2%)	
	IV	337 (22.7%)	132 (20.7%)	
T	1	462 (31.1%)	198 (31.1%)	0.0681
	2	164 (11.0%)	95 (14.9%)	
	3	690 (46.4%)	270 (42.4%)	
	4	170 (11.4%)	74 (11.6%)	
N	0	1,267 (85.3%)	561 (88.1%)	0.1989
	1	107 (7.2%)	44 (6.9%)	
	2	100 (6.7%)	29 (4.6%)	
	3	12 (0.8%)	3 (0.5%)	
M	0	1,392 (93.7%)	606 (95.1%)	0.1906
	1	94 (6.3%)	31 (4.9%)	
Radiotherapy	No	1,434 (96.5%)	614 (96.4%)	0.8987
	Yes	52 (3.5%)	23 (3.6%)	
Chemotherapy	No	1,137 (76.5%)	520 (81.6%)	0.0090
	Yes	349 (23.5%)	117 (18.4%)	

Table 2 provides an in-depth analysis of factors influencing CSS in the training cohort through both univariate and multivariate models, utilizing hazard ratios (HRs) and confidence intervals. In the univariate analysis, age emerged as a significant determinant of survival, with progressively higher risks in older age groups, culminating in the highest risk for those aged 80 and above (HR = 3.11, *p* < 0.001). Histological type also significantly affected outcomes; papillary TCC demonstrated a notably lower risk compared to TCC (HR = 0.49, *p* < 0.001). Cancer grade and stage were also strong predictors of outcomes, with higher stages and poorer differentiation associated with significantly increased risks. In the multivariate analysis, the results adjusted for confounding variables reinforced the findings from the univariate analysis. Older age groups continued to show a significantly increased risk, with the ≥80 age group having the highest risk (HR = 3.26, *p* < 0.001). Advanced cancer stages continued to show higher risks, with stage IV cancer exhibiting an HR of 7.39 (*p* < 0.001). Treatment modalities also impacted survival; while radiotherapy increased the risk of mortality (HR = 1.82, *p* = 0.001), chemotherapy was associated with a decreased risk (HR = 0.69, *p* < 0.001).

**Table 2:** Univariate and multivariate analysis of CSS in the training cohort

		Univariate	Multivariate
		HR (95% CI, *p*)	HR (95% CI, *p*)
Sex	Male	Reference	Reference
	Female	1.17 (1.00–1.36, *p* = 0.049)	1.15 (0.98–1.35, *p* = 0.078)
Race	White	Reference	
	Others	0.87 (0.68–1.12, *p* = 0.275)	
Age	<60	Reference	Reference
	60–69	1.67 (1.23–2.27, *p* < 0.001)	1.73 (1.28–2.35, *p* < 0.001)
	70–79	2.30 (1.73–3.05, *p* < 0.001)	2.20 (1.65–2.94, *p* < 0.001)
	≥80	3.11 (2.34–4.14, *p* < 0.001)	3.26 (2.42–4.40, *p* < 0.001)
Histology	Transitional cell carcinoma	Reference	Reference
	Papillary transitional cell carcinoma	0.49 (0.42–0.57, *p* < 0.001)	0.78 (0.66–0.92, *p* = 0.004)
Grade	Well differentiated	Reference	Reference
	Moderately differentiated	1.48 (0.75–2.91, *p* = 0.255)	1.62 (0.82–3.20, *p* = 0.165)
	Poorly differentiated	2.98 (1.57–5.64, *p* < 0.001)	2.21 (1.16–4.20, *p* = 0.016)
	Undifferentiated	2.78 (1.48–5.20, *p* = 0.001)	2.11 (1.12–3.99, *p* = 0.021)
Stage	I	Reference	Reference
	II	1.78 (1.28–2.47, *p* < 0.001)	1.69 (1.21–2.35, *p* = 0.002)
	III	2.54 (2.00–3.21, *p* < 0.001)	2.33 (1.83–2.97, *p* < 0.001)
	IV	7.26 (5.73–9.20, *p* < 0.001)	7.39 (5.64–9.69, *p* < 0.001)
T	1	Reference	
	2	1.68 (1.23–2.29, *p* = 0.001)	
	3	2.80 (2.27–3.47, *p* < 0.001)	
	4	7.64 (5.90–9.88, *p* < 0.001)	
N	0	Reference	
	1	3.24 (2.55–4.13, *p* < 0.001)	
	2	3.10 (2.41–3.98, *p* < 0.001)	
	3	5.66 (3.10–10.31, *p* < 0.001)	
M	0	Reference	
	1	6.57 (5.17–8.34, *p* < 0.001)	
Radiotherapy	No	Reference	Reference
	Yes	2.89 (2.09–4.00, *p* < 0.001)	1.82 (1.29–2.58, *p* = 0.001)
Chemotherapy	No	Reference	Reference
	Yes	1.27 (1.07–1.51, *p* = 0.007)	0.69 (0.56–0.85, *p* < 0.001)

**Figure 2:**
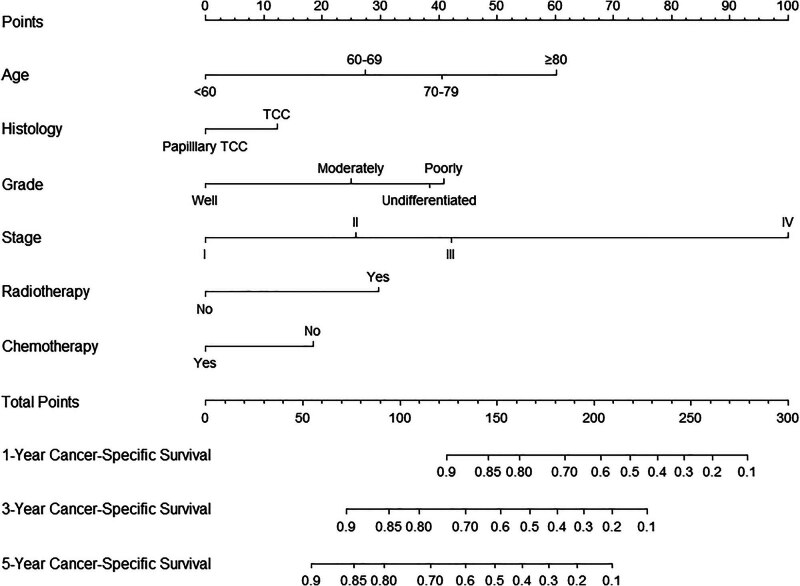
Nomogram for predicting 1-year, 3-year, and 5-year CSS rates in renal pelvic cancer patients after surgery.

**Figure 3:**
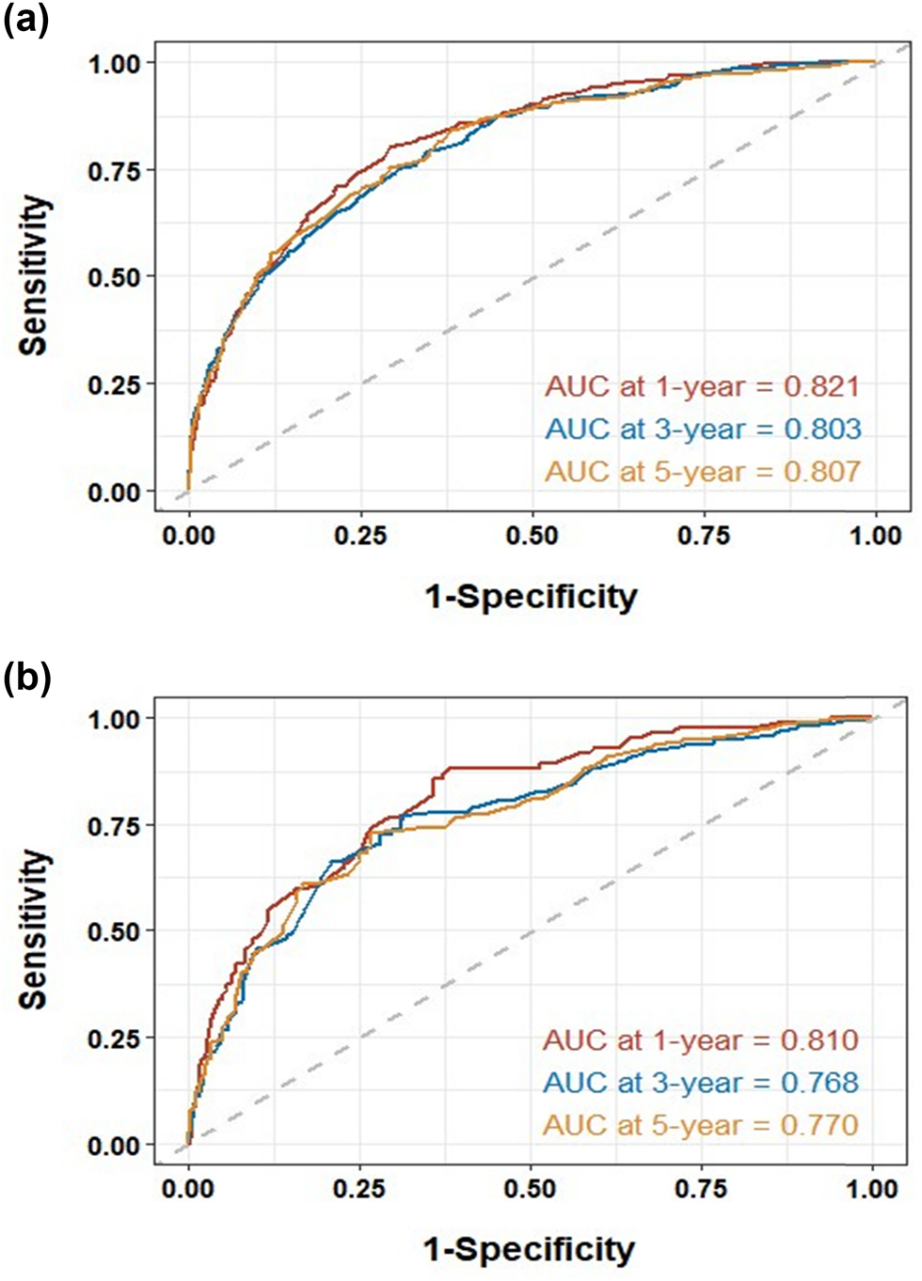
Analysis of the ROC curve for the nomogram in predicting patient prognosis in the training and validation cohorts. The AUC for 1-year, 3-year, and 5-year predictions in the training (a) and validation cohorts (b).

**Figure 4:**
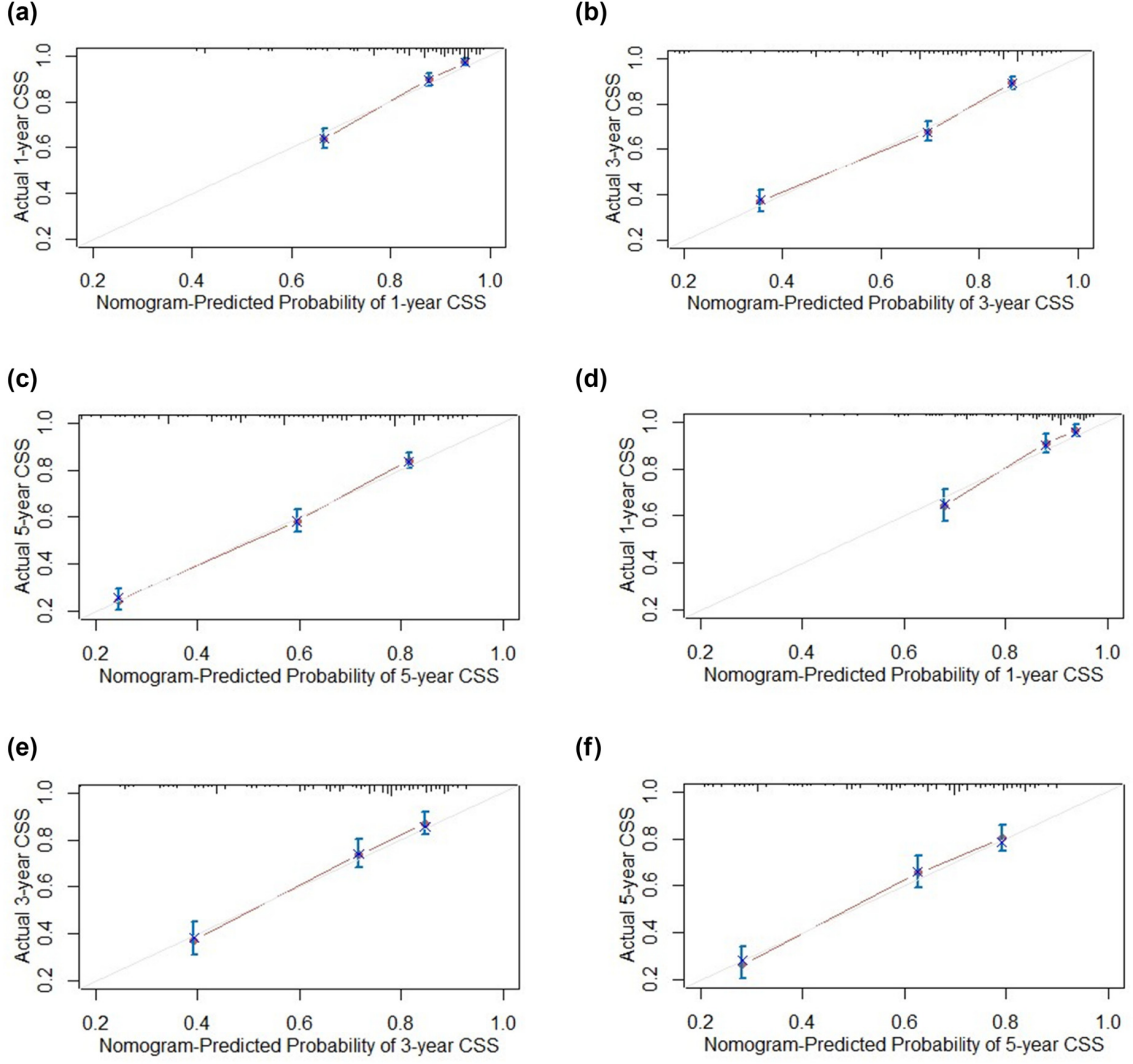
The calibration plots for predicting CSS in the training cohort at 1-year (a), 3-year (b), and 5-year (c), and in the validation cohort at 1-year (d), 3-year (e), and 5-year (f). Actual CSS is plotted on the *y*-axis; nomogram-predicted probability of CSS is plotted on the *x*-axis.

**Figure 5:**
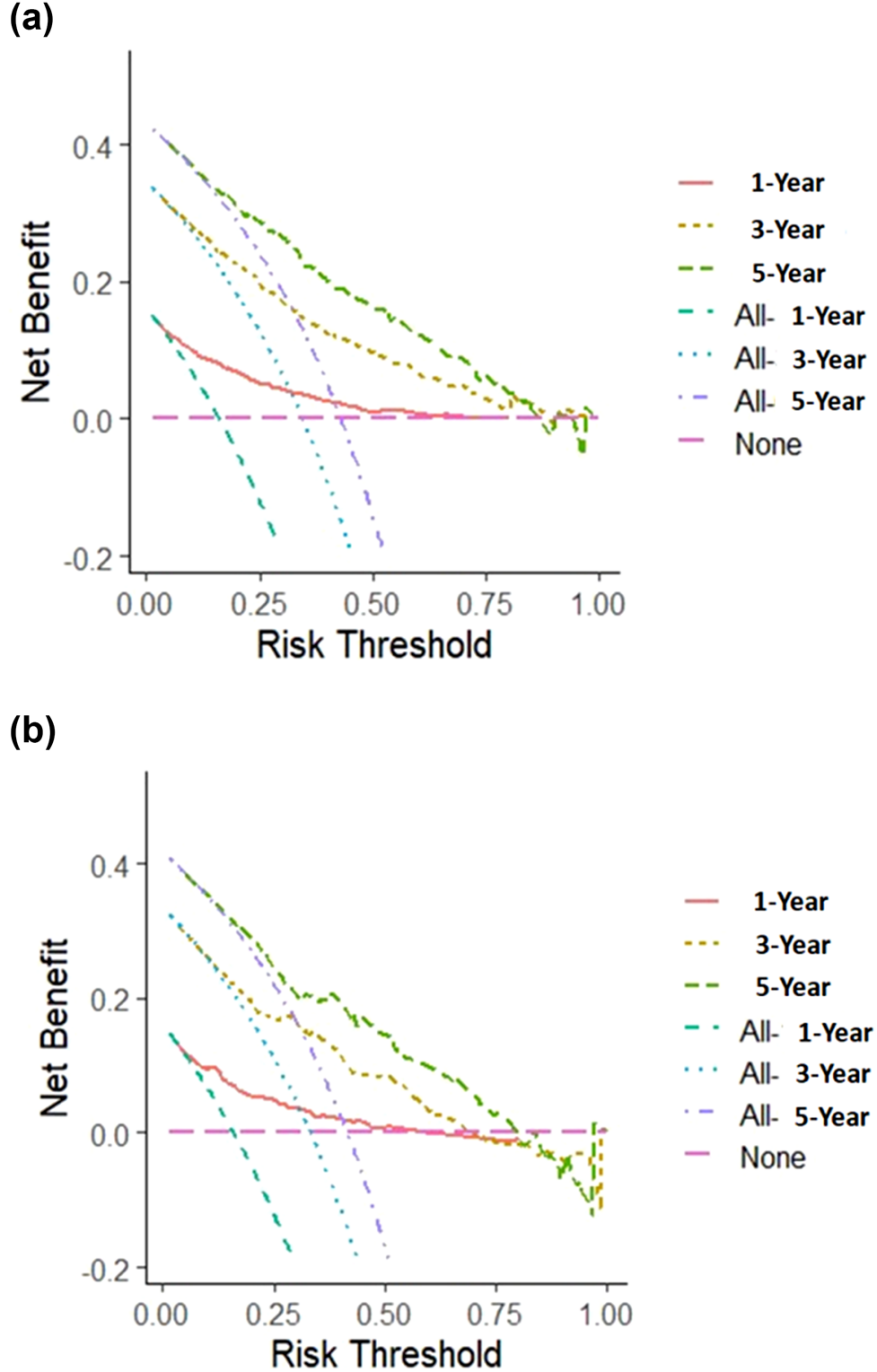
DCA of the nomogram for predicting 1-year, 3-year, and 5-year CSS in the training (a) and validation cohorts (b). The *x*-axis is the threshold probability, and the *y*-axis is the net benefit.

Given that staging is derived from the TNM classification, to simplify the nomogram, only the stage was incorporated. Consequently, the variables used to construct the nomogram included age, histology, grade, stage, radiotherapy, and chemotherapy (C-index: 0.746) (Figure 2). To use the nomogram, each variable (e.g., age, tumor histology, grade, stage, radiotherapy, chemotherapy) corresponds to a specific point value on the top “Points” axis. For a given patient, draw a vertical line from the variable value to the Points axis to obtain a score for that variable. After repeating this for all variables, sum the individual points to obtain a Total Points value at the bottom. Then, draw a vertical line down from the Total Points axis to estimate the 1-, 3-, and 5-year CSS probabilities. The analysis of the ROC curves for the nomogram, as used to predict patient prognosis in the training and validation cohorts, is presented in Figure 3. The area under the curve (AUC) for 1-, 3-, and 5-year predictions in the training cohort were 0.821, 0.803, and 0.807, respectively. Correspondingly, the AUC values for the validation cohort were 0.810, 0.768, and 0.770, indicating robust predictive accuracy across both cohorts. The calibration plots for predicting 1-, 3-, and 5-year CSS in the training and validation cohorts are depicted in Figure 4. These plots demonstrate the accuracy of the predictions by comparing the observed outcomes with the probabilities forecasted by the model. DCA of the nomogram for predicting 1-, 3-, and 5-year CSS in the training and validation cohorts is illustrated in Figure 5. This analysis underscores the clinical usefulness of the nomogram by demonstrating its net benefits across various risk thresholds.

## 4 Discussion

Currently, there are few nomograms available for predicting outcomes following surgery for renal pelvis cancer. To address this gap, we developed and subsequently validated a nomogram. Our nomogram demonstrated a C-index of 0.746, reflecting moderate to good discriminatory performance. This result aligns closely with previously published prognostic models for UTUC. For example, Wang et al. [12] reported C-indices of 0.740 and 0.729 for high-grade UTUC in the training and internal validation cohorts, respectively, while models for low-grade disease yielded slightly lower values ranging from 0.714 to 0.731. Furthermore, well-established postoperative nomograms, such as that analyzed by Pallauf et al. [13], have shown similar predictive accuracy. Collectively, these comparisons underscore the competitive performance and clinical relevance of our nomogram within the context of existing UTUC prediction tools.

In the current study, no significant differences were observed between men and women regarding CSS rates in renal pelvic cancer. A study conducted by Deuker et al. [14] on the metastatic sites of UTUC reported that the lungs were the most frequent metastatic sites for both men and women, with incidences of 28.2 and 26.4%, respectively. This was followed by bone metastases in men (22.3%) and liver metastases in women (24.4%), compared to 18.0 and 20.5% in the opposite sexes, respectively. Despite these findings, the limitations of our study prevent us from confirming these results conclusively. Nevertheless, our data indicate metastatic rates of 5.69% in men and 6.16% in women. Age has been identified as a significant determinant of CSS for renal pelvic cancer in the present study. Research conducted by Eismann et al. [15] demonstrated that median survival durations following radical nephroureterectomy for UTUC were differentiated by age groups: under 75 years at 115 months, 75–79 years at 55 months, 80–84 years at 28 months, and 85 years and older at 20 months. This raises a pertinent query regarding whether elderly patients with UTUC may be receiving overly aggressive treatment via radical nephroureterectomy. Further analysis revealed that there was a notable 5-year OS advantage for patients with an Eastern Cooperative Oncology Group (ECOG) performance status score of 0, who exhibited an OS rate of 82.3%, in contrast to scores of 1 and 2, which showed significantly lower OS rates of 46.0 and 15.0%, respectively (*p* < 0.001). These findings suggest that the ECOG performance status should be a critical consideration prior to undertaking radical nephroureterectomy for UTUC.

Numerous researchers have investigated the influence of variant histology on the prognosis of UTUC. In a study involving 128 patients who underwent surgery for this condition, Artykov et al. [16] observed that variant histology was present in 14.8% of the cases. Their findings revealed no statistically significant association between variant histology and both CSS (*p* = 0.514) and OS (*p* = 0.515) rates. However, the limited number of patients in this study may constrain the generalizability of these results. In a cohort of 11,809 patients with upper urinary tract tumors, Deuker et al. [17] identified 154 cases (1.3%) of squamous cell carcinoma (SCC), 86 cases (0.7%) of adenocarcinoma, 39 cases (0.3%) of neuroendocrine carcinoma, and 38 cases (0.3%) of other variant histologies. Additionally, their analysis revealed that, across all stages, cancer-specific mortality was higher for patients with SCC compared to those with UTUC. Tully et al. [18] presented a study involving a large cohort of patients with variant histologies of tumors in the renal pelvis. The study encompassed 826 patients diagnosed with various variant histologies, including adenocarcinoma in 298 cases, SCC in 291 cases, sarcomatoid carcinoma in 137 cases, and other histologies in 100 cases. Their findings indicated that patients with adenocarcinomas exhibited longer OS, with a HR of 0.76, whereas those with sarcomatoid carcinomas demonstrated shorter OS, with a HR of 1.59, compared to patients with pure UC. Given that TCC is the predominant histological type in renal pelvic cancer, our study exclusively included TCC and its variant, papillary TCC. We observed a significant difference in CSS between these two subtypes.

In a previous investigation, Langner et al. [19] reported that tumor grade in UTUC did not significantly influence metastasis-free survival, as indicated by a p-value of 0.06. This finding was drawn from a cohort of 190 cases, analyzed using a two-tiered grading system. In a study conducted in Taiwan involving 141 patients with UTUC, including 71 cases in the renal pelvis and 70 in the ureter, Tan et al. [20] reported that tumor grade was a significant predictor of 5-year survival for renal pelvis UC but not for ureteral UC, as determined through multivariate logistic regression analysis. Despite the relatively small sample size of 141 patients, the findings underscore the importance of considering the impact of tumor grade on different tumor sites. Perez-Montiel et al. [21] reported that primary UC of the renal pelvis is predominantly of high histologic grade. This observation aligns with our findings, where the proportion of Grade III tumors (23.1%) exceeded that of Grade II (11.7%) and Grade I (3.2%). Due to the propensity of UC of the renal pelvis to frequently exhibit unusual morphologic features, the authors suggested that the presence of a high-grade UC should consistently be considered when evaluating tumors in the renal pelvis that display atypical morphological characteristics.

In their study of ureteral and pelvic cancer UC, Tan et al. [20] identified tumor stage as a significant predictor of outcomes in multivariate logistic regression analyses. Kim et al. [22] demonstrated that disease progression-free survival in UTUC is significantly associated with pathological T3 and T4, reporting a HR of 13.3. Zhai et al. [23] found that lymph node dissection was linked to improved OS and CSS in patients with UTUC at pathological stages T3 and T4, based on multivariable Cox regression analyses. They recommended considering lymph node dissection for patients at these stages. Our study confirms that stage is a critical predictive factor for CSS. Given that the stage is composed of TNM classifications, and to simplify the nomogram under conditions where T, N, and M categories vary, we included only the overall stage in the development of the nomogram.

The apparent adverse effect of chemotherapy observed in the univariate analysis (HR = 1.27) was no longer significant in the multivariate model, likely due to confounding by indication. Chemotherapy is more commonly administered in patients with advanced or high-risk disease, who inherently have poorer prognoses. After adjusting for stage and other covariates, the independent effect of chemotherapy may be obscured. This highlights the need for cautious interpretation of treatment variables in observational studies without randomization.

This retrospective study is subject to several limitations inherent in its design. First, one significant constraint is the absence of lifestyle data, notably smoking habits. Ross et al. [24] highlighted that prolonged cigarette smoking (over 25 years) significantly elevates the risk of developing renal pelvis and ureter cancers, with smokers experiencing a relative risk of 4.5 compared to non-smokers (*P* < 0.0001). Second, the unavailability of comorbidity data in the SEER database may introduce bias into the study outcomes. The absence of these covariates may introduce residual confounding, and future validation using datasets that incorporate such variables is warranted to improve prognostic accuracy. Third, SEER lacks detailed surgical procedural codes distinguishing between radical and conservative approaches. Therefore, we were unable to stratify patients by surgical technique. Given the potential impact of surgical extent on outcomes, future studies incorporating institutional or multi-center datasets with more detailed operative information are needed to refine prognostic modeling and enhance clinical applicability. Fourth, a key limitation of this study is the absence of external validation using a non-SEER cohort. While internal validation demonstrated robust predictive performance, future studies should aim to validate this nomogram in independent patient populations to enhance its generalizability and clinical utility. Despite these limitations, the SEER database provides information on patient demographics, tumor morphology, stages at diagnosis, initial treatment approaches, and survival outcomes. The nomogram development from the extensive data enables the derivation of robust evidence-based insights, which are crucial for clinical decision-making.

## 5 Conclusion

Age, histology, grade, and stage were identified as significant risk factors influencing CSS in patients diagnosed with renal pelvic UC after surgical intervention. The construction of the nomogram, utilizing selectively chosen factors from extensive datasets, facilitates the generation of robust, evidence-based insights that are crucial for informed clinical decision-making.
